# From Grazing Resistance to Pathogenesis: The Coincidental Evolution of Virulence Factors

**DOI:** 10.1371/journal.pone.0011882

**Published:** 2010-08-11

**Authors:** Sandrine Adiba, Clément Nizak, Minus van Baalen, Erick Denamur, Frantz Depaulis

**Affiliations:** 1 Laboratoire d'Ecologie, CNRS UMR7625, Université Pierre et Marie Curie, Paris Universitas, Paris, France; 2 Laboratoire de Spectrométrie de Physique, CNRS UMR5588, Grenoble, France; 3 INSERM U722 and Université Paris Diderot, Paris, France; 4 Laboratoire d'Ecologie, CNRS UMR7625, Ecole Normale Supérieure, Paris, France; University of Hyderabad, India

## Abstract

To many pathogenic bacteria, human hosts are an evolutionary dead end. This begs the question what evolutionary forces have shaped their virulence traits. Why are these bacteria so virulent? The coincidental evolution hypothesis suggests that such virulence factors result from adaptation to other ecological niches. In particular, virulence traits in bacteria might result from selective pressure exerted by protozoan predator. Thus, grazing resistance may be an evolutionarily exaptation for bacterial pathogenicity.

This hypothesis was tested by subjecting a well characterized collection of 31 *Escherichia coli* strains (human commensal or extra-intestinal pathogenic) to grazing by the social haploid amoeba *Dictyostelium discoideum*. We then assessed how resistance to grazing correlates with some bacterial traits, such as the presence of virulence genes. Whatever the relative population size (bacteria/amoeba) for a non-pathogenic bacteria strain, *D. discoideum* was able to phagocytise, digest and grow. In contrast, a pathogenic bacterium strain killed *D. discoideum* above a certain bacteria/amoeba population size. A plating assay was then carried out using the *E. coli* collection faced to the grazing of *D. discoideum*. *E. coli* strains carrying virulence genes such as *iroN*, *irp2*, *fyuA* involved in iron uptake, belonging to the B2 phylogenetic group and being virulent in a mouse model of septicaemia were resistant to the grazing from *D. discoideum*. Experimental proof of the key role of the *irp* gene in the grazing resistance was evidenced with a mutant strain lacking this gene. Such determinant of virulence may well be originally selected and (or) further maintained for their role in natural habitat: resistance to digestion by free-living protozoa, rather than for virulence *per se*.

## Introduction

The evolution and the maintenance of virulence are among the most important topics addressed in recent years by evolutionary biologists. Different theories have been proposed and become the focus of intense debate. The “conventional wisdom” [1] known also as the “avirulence hypothesis” suggests that parasites would always evolve to become avirulent. Virulence is then considered as a maladaptation of new or recent associations between parasites and hosts. This hypothesis was challenged during the 1980's. A number of theories [2], [3], [4], [5], [6], [7] proposed that virulence would be maintained by natural selection and should depend on the mechanism of transmission. This “trade-off” hypothesis between virulence and transmission is still investigated [8]. Two alternative models: short-sighted within host-selection and the coincidental evolution are two other ways by which natural selection can favour virulence, irrespective of any relationship between virulence and transmission. Our work will focus on the last hypothesis: the coincidental evolution which hypothesizes that virulence is a coincidental by-product of the adaptation to other ecological niches.

It is well known that free living protozoa may affect bacterial populations in multiple ways. They may act as predators but also provide a protective environment and even act as vectors [9]. Their long co-evolutionary history suggests that a series of adaptations ensuring bacterial survival should have emerged. Moreover, mechanisms that improve resistance to digestion by predators such as free-living amoebae may well express themselves as virulence factors in other organisms [10]. Thus bacterial traits such as adherence, digestion resistance, intracellular toxin production and intracellular replication [11], [12] or outer-membrane structures [13] might facilitate a transition from free-living organism to intracellular parasite. The ability of bacteria to resist and exploit a normally hostile niche to ensure survival was discovered in the bacteria *Legionella pneumophila*, which is able to multiply within certain species of free-living amoeba [14]. The ability of *L. pneumophila* to parasitize macrophages and cause human disease may thus well be a consequence of its resistance to amoebae. For many diseases such as Legionnaire disease, Lyme disease, pneumonia disease caused by Hantavirus, human play no (or at best negligible) role in the transmission and are then an evolutionary dead end [15]. Genes that cause these effects must therefore be favoured by their effects elsewhere. These cases where virulence is obviously of no selective value (neutral) [16] led to the hypothesis of coincidental evolution of virulence factors. Adaptation to commensal and/or saprophyte habitats may thus coincidentally promote some virulence factors rather than selection for virulence *per se* [17]. However, the coincidental evolution hypothesis has not been tested.

*Escherichia coli* is a good biological model to investigate such coincidental evolution. This Gram-negative and facultatively anaerobic bacterium occurs both as commensal and pathogenic strains. Commensal *E. coli* (most strains) use their host as a suitable habitat without any noteworthy benefit or detriment for the host, whereas pathogenic strains may cause harm or even death [18]. Even so-called extra-intestinal pathogenic *E. coli* (ExPEC) strains found in mammal intestinal tracts do not normally cause disease but they do when they incidentally invade sterile niches such as urinary tract, blood [19], [20] or cerebrospinal fluid. Besides this primary habitat, *E. coli*'s secondary habitat involves the external environment: water, sediment, and soil [21], [22], [23] where some specific strains may grow [24], [25], [26].

*E. coli* population genetic structure is mainly clonal, with four main phylogenetic groups denoted A, B1, B2 and D [27], [28]. Each phylogenetic group shows various life history traits and differs in its phenotypic and genotypic characteristics [27], [29]. Compared to commensal strains, ExPEC have additional virulence factors such as adhesins (e.g. P and I fimbriae), iron-acquisition systems (e.g. aerobactin), host defence-avoidance systems (e.g., capsule, lipopolysaccharide), and toxins (e.g., hemolysin, cytotoxic necrotizing factor1) [30], [31]. ExPEC virulence factors, encoded by genes often clustered on so-called pathogenicity island (PAI), are mainly found in strains of the phylogenetic group B2 and D but scarcely in the phylogenetic groups A and B1 to which the majority of commensal and extraintestinal pathogenic *E. coli* strains belong [32], [33], [34]. The maintenance and the evolution of these genes associated with virulence within the phylogenetic group B2 suggest that virulence could be a coincidental by-product: these genes are implicated in complex commensal niche colonization [15], [35] where bacteria are subjected to grazing by protista such as free-living amoeba.

In a similar fashion, amoebae that occur in the human intestinal tract (*Entamoeba* species) [36], [37], [38] typically also occur in external environment too, in habitats such as fresh water, moist soil, at various interfaces (water-soil, water-plant, water-air) [39]. They are distributed worldwide, but the set of species in a particular location depends on abiotic factors and food availability. Amoebae are particularly effective bacterial predators. The group of free-living amoeba is the only group of organisms that can cause a decrease of a bacterial population from 10^8^ down to 10^5^ per gram of soil [40], [41], [42]. *Dictyostelium discoideum*, a haploid social amoeba has been used as a model to study host-factors involved in cellular aspects of host-pathogen interactions [43], [44]. The common mechanisms of infection needed to infect both mammals and *D. discoideum* by a wide variety of pathogens reinforces the use of the *E. coli/D. discoideum* system as a valid model to study host-pathogen relations and their evolution [43], [45]. In addition, elaborate genetic tools including genomic sequence [46] and plasmids allowing constitutive or inducible expression of genes are now available.

In the context of the variety of ecological and evolutionary factors involved in the interconnected life histories of bacteria and amoeba, we investigated here evolutionary forces selecting and maintaining virulence factors. To test the coincidental evolution hypothesis, a first goal was to test whether ecological interactions between *D. discoideum* and *E. coli* have an effect. The first experiment details the relative population size effect (bacteria/amoeba) on amoeba grazing resistance. Secondly, the ability for pathogenic bacteria to kill or inhibit amoeba growth was assessed. Our main experiment compares the grazing resistance of a collection of 31 human commensal and pathogenic *E. coli* strains to predation by the social amoeba *D. discoideum* and assesses the correlation of resistance with known virulence genes. The results we obtained suggested that the so-called High pathogenicity island (HPI) of *E. coli* plays an important role. This role was further assessed using a mutant bacterial strain with inactivated genes and its complemented isogenic strain.

## Results

### Relative population size effect on grazing resistance

To test whether the relative population size of *E. coli* and amoeba cells affect the grazing of bacteria by amoeba, plating assay was performed. *D. discoideum* cells were plated on HL5 agar plates with either non-pathogenic *E. coli* strain REL606 or pathogenic *E. coli* strain 536. We tested different bacteria/amoebae cells ratio by measuring grazing capacity at different bacteria (vs amoebae) population sizes for a given amoeba (vs bacteria) population size (summing up to 60 plates). Over the course of a few days, the bacteria formed lawns on these plates with amoeba embedded in them. The bacteria phagocytized by *D. discoideum* were assessed through the occurrence of bacterial lysis plaques.

**Figure 1:**
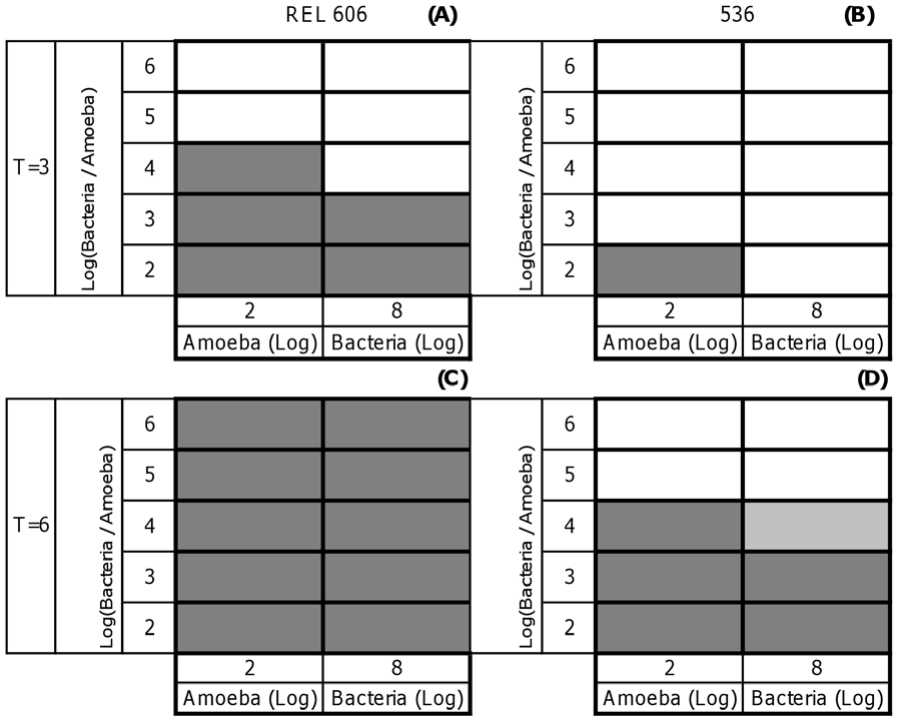
Relative population size effect on *E. coli*/*D. discoideum* interactions. Bacterial lysis plaques (strains REL 606 and 536) observed three days (T = 3) or six days (T = 6) after plating. Dark gray color represents three replicates with bacterial lysis plaques. Gray color represents one replicate with bacterial lysis plaques. White color represents zero replicate with bacterial lysis plaques. (A) Three days after plating, strain REL 606. (B) Three days after plating, strain 536. (C)Six days after plating, strain REL 606. (D) Six days after plating, strain 536.

Three days after plating, with 10^2^ amoeba cells (Figure 1 (A)) plaques were observed for three REL 606 bacterial population sizes: 10^4^,10^5^,10^6^ cells and one population size of strain 536: 10^4^ cells (Figure 1 (B)). In the symetrical experiment (10^8^ bacteria cells), we observed plaques only with the strain REL606 for two amoeba population sizes 10^6^ and 10^5^ (Figure 1 (A)) and not for the strain 536 (Figure 1 (B)).

Six days after plating, plaques were noticed with the strain REL606 for both ways of the experiments and for all population sizes (bacteria vs amoebae) (Figure 1 (C)). But for the pathogenic strain 536, plaques appeared for three relative population sizes: 10^2^, 10^3^ and 10^4^ bacteria/amoeba (Figure 1 (D)). In the symetrical experiment, with 10^4^ amoebae, plaques were observed for a single replicate (Figure 1 (D)).

These results show that with the pathogenic strain 536, up to a relative population size of 10^4^, bacterial lysis plaques arise, but not with greater relative population sizes.

*D. discoideum* feeds on *E. coli* REL606 whatever the relative population size (Figure 1 (C)) and form plaques that can be noticed after six days. *D. discoideum* does not form plaques on the lawns of *E. coli* 536 for a relative population size greater than 10^4^ bacteria/amoeba (strain effect: χ^2^ = 4.945, *p* = 0.0262; population size effect: χ^2^ = 4.04, *p* = 0.0444; strain* population size effect: χ^2^ = 4.04, *p* = 0.0444). Therefore, in contrast with *E. coli* REL606, *E. coli* 536 resists digestion by amoebae. *E. coli* 536 may kill amoeba or at least substantially inhibit their growth.

### Death versus growth inhibition

To test the ability of the pathogenic strain *E. coli* 536 to kill *D. discoideum* or inhibit its growth, bacteria (the pathogenic strain 536 and the non-pathogenic REL606) were plated with amoeba at 10^6^ relative population size. Two and six days after plating, the top layers were transferred in a MCPB liquid medium. This liquid culture was then screened with BacLight kit in which living, healthy cells fluoresce in green whereas damaged cells show up red [26].

**Figure 2:**
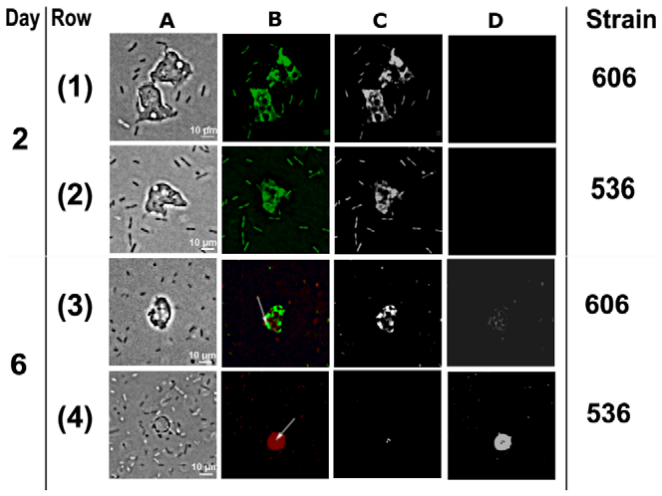
Visualization of the *E. coli/D. discoideum* viability. (A) Phase contrast microscopy (20×). (B) Combined Red and Green: Green fluorescent stain reflect living cells, red fluorescence reflect cells with damaged membranes. (C) Green channel alone. (D) Red channel alone: (1) Amoeba with the strain *E. coli* B REL 606 two days after plating, all cells are green reflecting living cells. (2) Amoeba with the strain *E. coli* 536 two days after plating, all cells are green reflecting living cells. (3) Amoeba with the strain *E. coli* B REL 606 six days after plating, red fluorescent bacteria are cells with damaged membranes (white arrow), green fluorescent cells are alive (amoeba and bacteria). (4) Amoeba with the strain *E. coli* 536 six days after plating, green fluorescent bacteria are alive cells (white arrow), red fluorescent amoeba and bacteria are cells with damaged membranes.

Two days after plating, both bacteria (536 and REL606 strains) and amoeba fluoresce in green (Figure 2 (B) first and second rows) indicating living cells. Six days after plating, for the experiment with strain REL606, we observed some bacteria with green and red fluorescent colour outside and inside amoeba (white arrow), red fluorescence indicating membrane cell damages. Amoeba in green fluorescent staining (Figure 2 (B) third row) indicated cell alive. Thus, *D. discoideum* remained alive and had digested some *E. coli* 606 bacteria cells. Similar results were observed with the strain B/r (data not shown). The culture with *E. coli* REL606 thus revealed a high amoeba density which implies that amoeba can achieve high growth rates in this diet. In contrast, six days after plating, the picture with the pathogenic strain *E. coli* 536 showed three or more living *E. coli* cells in the *D. discoideum* one (Figure 2 (B) fourth row) and other bacteria outside the amoeba in red fluorescence. Staining with BacLight kit revealed green fluorescent, living bacteria (white arrow) inside amoeba. Red fluorescent staining indicated damaged membranes of the *D. discoideum* that should lead to cell death. Similar results were obtained using pathogenic strain IAI51 (data not shown). This fluorescent straining showed living *E. coli* 536 bacteria cells and *D. discoideum* dead cells.

### Grazing resistance

**Figure 3:**
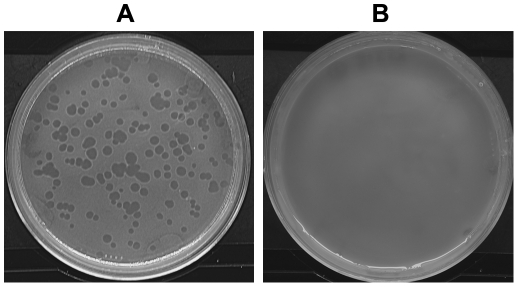
Bacterial lysis plaque occurrence. (A) A non-virulent *E. coli* strain (B REL606; 10^8^ cells) was plated with *D. discoideum* (10^2^ cells). Bacterial lysis plaques were observed, characteristic of the grazing phenotype. (B) A virulent *E. coli* strain (536; 10^8^ cells) was plated with *D. discoideum* (10^2^ cells). No bacterial lysis plaques were observed, characteristic of the grazing resistance phenotype.

In order to compare the ability of pathogenic *E. coli* strains to resist grazing protozoa with that of commensal strains and to identify bacterial virulence traits that could have been favoured and maintained by selection for other functions than virulence *per se*, a plating assay was performed for each strain of the bacterial collection (including pathogenic and commensal ones) at a low (10^4^) and a high (10^8^) initial bacterial population size with three amoeba population sizes (10, 10^2^, 10^3^). Three replicates were run for each combination (N = 558 plates). A few days after plating, the bacteria formed lawns on these plates in which the amoebae were embedded. At low bacterial population size (10^4^), in agreement with the previous experiments, *D. discoideum* formed plaques on lawns with all *E. coli* strains. At high bacterial population size (10^8^), *D. discoideum* fed on some *E. coli* strains but for some other strains, did not form any bacterial lysis plaques (Figure 3). The results were highly reproducible with identical results in all replicates for the strains IAI51, IAI52, B REL606, RS218 and B/r.

### Association between grazing resistance and bacterial virulence traits

**Figure 4:**
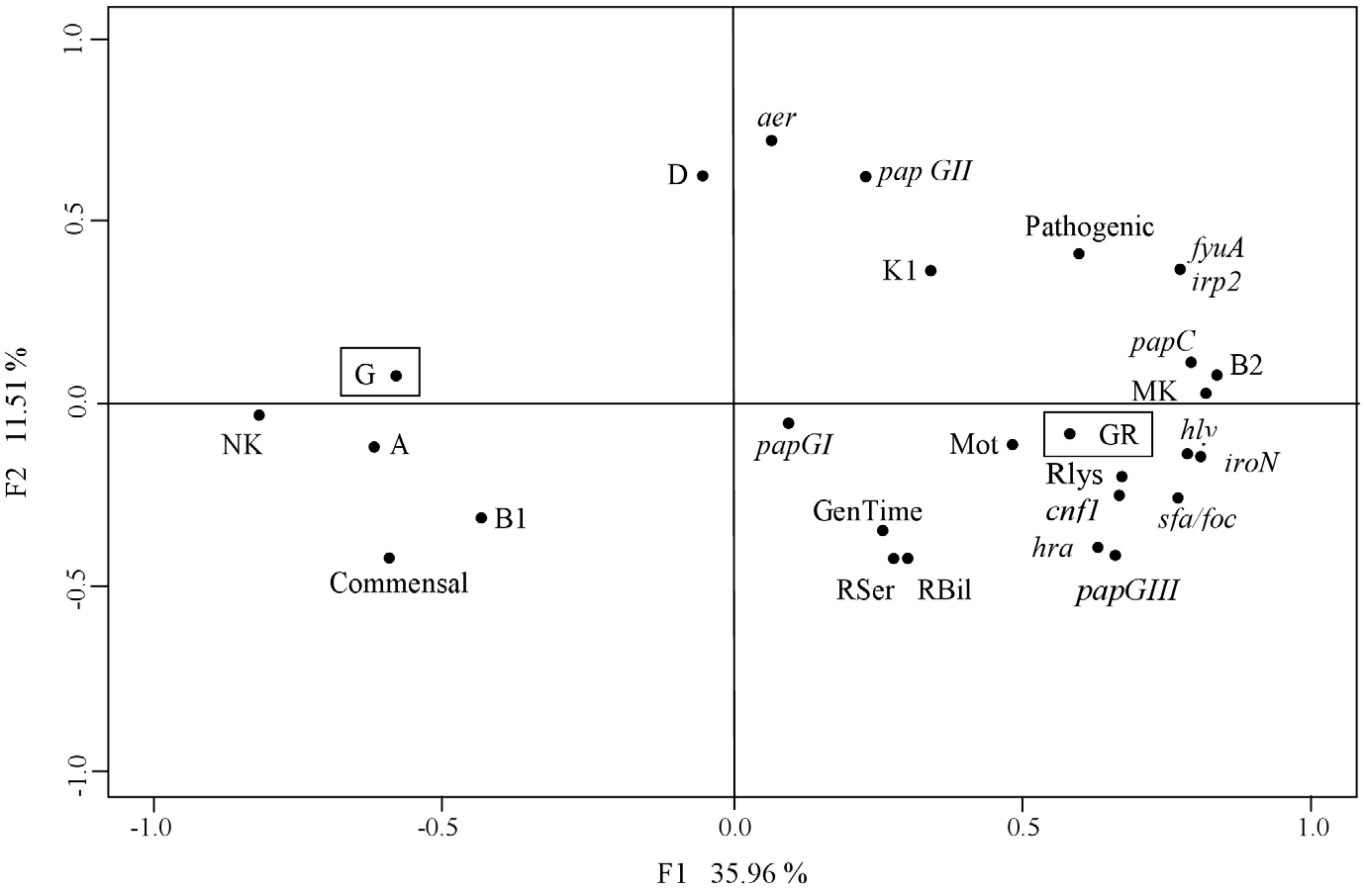
Factorial analysis of correspondences. Projection on the F1/F2 plane of the 28 bacterial traits characterized for the 31 *E. coli* collection strains. A, B1, B2 and D: phylogenetic groups; Com: commensal, Path: pathogenic; *papGI*, *II*, *III: papG* and corresponding three existing alleles; K1: K1 antigen (capsule), *sfa/foc*, *iroN*, *hly*, *cnf1*, *hra*, *fyuA*, *irp2*, *aer*: virulence genes; RSer: Serum resistance; RBil: Bile resistance; RLys: lysozym/lactoferrin resistance; Mot: motility; GenTim: generation time; K: mouse killer, NK: mouse non-killer; G: grazing, GR: grazing resistance (boxed). The projection of the variables onto the F1/F2 plane accounts for 47.47% of the total variance. The first group (F1 negative values and F2 positive and negative values) encompasses following traits: the grazing, the commensal origin, the absence of mouse lethality and the phylogenetic groups A, B1 and D. The second main group (F1 positive values and F2 positive and negative values) encompasses the grazing resistance trait, the mouse lethality, the pathogenic origin of the strains, the phylogenetic group B2, virulence genes, the resistance to serum, bile and lysozyme/lactoferrin, the motility and fast growth rate.

To identify associations between grazing resistance to protozoa and various bacterial traits, a FAC (Factorial Analysis of Correspondence) was applied using all data available for the collection of strains (Table 1). The FAC (Figure 4) is based on χ^2^ distances using a covariance matrix. It detects positive and negative associations existing and measures the contribution of each factor to the total inertia. The projection of the variables onto the F1/F2 plane accounts for 47.47% of the total variance and distinguishes two main groups of variables by positive and negative values for the first factor F1. The first group (F1 negative values and F2 positive and negative values) encompasses the following traits: grazing, commensal origin, the absence of mouse lethality and the phylogenetic groups A, B1 and D. The second main group (F1 positive values and F2 positive and negative values) encompasses grazing resistance trait, mouse lethality, pathogenic origin of the strains, phylogenetic group B2, virulence factors, the resistances to serum, bile and lysozyme/lactoferrin, the motility and fast growth rate.

**Table 1:** Phenotypic and genotypic characteristics of the *E. coli* strains studied.

			Virulence gene b											Phenotype c					Model tested d	
	Strain ID	Phylogenetic groups a	K1	*sfa/foc*	*iroN*	*aer*	*papC*	*papG*	*hly*	*cnf1*	*hra*	*fyuA*	*irp2*	RSer	RLysoz	RBil	Mot	GenTim	Mouse	Amoeba
Laboratory adapted strain	K-12 MG1655	A	−	−	−	−	−	−	−	−	−	−	−	−	−	−	−	−	NK	G
	B/r		ND	ND	ND	ND	ND	ND	ND	ND	ND	ND	ND	ND	ND	ND	ND	ND	ND	G
	B REL 606	A	ND	ND	ND	ND	ND	ND	ND	ND	ND	ND	ND	ND	ND	ND	ND	ND	ND	G
Commensal strain	Ben13f	B2	−	+	+	−	+	III	−	−	+	+	+	+	−	+	−	+	K	GR
	ColF6c	B2	−	−	−	−	+	II	+	+	+	+	+	+	+	+	−	+	K	GR
	ECOR58	B1	−	+	+	−	−	−	−	−	−	−	−	+	+	+	+	+	K	GR
	ED1a	B2	−	−	−	+	−	−	−	−	−	+	+	−	−	−	−	−	NK	GR
	IAI1	B1	−	−	−	−	−	−	−	−	−	−	−	+	−	+	−	+	NK	GR
	IAI12	A	−	−	−	−	−	−	−	−	−	−	−	+	−	+	−	+	NK	G
	IAI13	A	−	−	−	−	−	−	−	−	+	−	−	−	−	−	−	−	NK	G
	IAI15	A	−	−	−	−	−	−	−	−	−	+	+	−	−	−	+	−	NK	G
	IAI2	B1	−	−	−	−	−	−	−	−	−	−	−	−	−	−	−	−	NK	G
	IAI4	A	−	−	−	−	−	−	−	−	−	−	−	−	−	+	−	+	NK	G
Pathogenic strain	536	B2	−	+	+	−	+	III	+	−	+	+	+	+	+	+	+	+	K	GR
	CFT073	B2	−	+	+	+	+	II	+	−	−	+	+	+	+	−	+	−	K	GR
	EC7372	B2	−	−	−	+	+	II	+	−	−	+	+	+	−	+	+	+	K	GR
	ECOR64	B2	−	+	+	−	−	−	−	−	−	+	+	+	+	−	−	−	K	GR
	F11	B2	−	+	+	−	+	III	+	+	+	+	+	+	+	+	+	+	K	GR
	F63	B2	+	+	+	−	+	III	+	+	+	+	+	+	+	+	+	−	K	GR
	IAI19	A	−	−	−	−	−	−	−	−	−	−	−	+	+	−	+	−	NK	G
	IAI21	B1	−	−	−	−	−	−	−	−	−	−	−	−	−	−	−	−	NK	G
	IAI39	D	+	−	−	+	+	II	−	−	−	+	+	−	−	−	−	−	K	G
	IAI44	A	−	−	−	−	−	−	−	−	−	+	+	−	−	−	+	−	NK	GR
	IAI48	B2	−	−	−	+	−	−	−	−	−	−	−	+	−	+	−	−	NK	GR
	IAI49	B2	−	−	+	−	−	−	−	−	−	+	+	+	+	−	+	+	K	GR
	IAI51	B2	+	+	+	−	−	−	−	−	−	+	+	−	+	−	−	+	K	GR
	IAI52	B2	+	+	+	−	−	−	−	−	−	+	+	−	+	−	−	+	K	G
	IAI60	B2	+	−	+	+	+	II	−	−	−	+	+	−	−	+	+	+	NK	GR
	IAI64	B2	−	+	+	−	+	III	+	+	+	+	+	−	−	−	+	+	K	G
	IAI72	B2	−	+	+	+	+	II	+	+	+	+	+	−	+	+	−	−	K	GR
	IAI73	B2	−	+	+	−	+	III	+	+	+	+	+	+	+	+	+	−	K	GR
	IAI74	B2	+	+	+	−	+	III	+	+	+	+	+	−	+	−	+	+	K	GR
	J96	B2	−	+	+	−	+	I+III	+	+	+	+	+	−	+	+	+	−	NK	G
	RS218	B2	+	+	+	−	+	III	+	+	+	+	+	+	+	+	+	+	K	GR

a Phylogenetic groups determined as in [74].

b Virulence genes determined as in [73], (+) presence, (−) absence. All these data are from [72] except the amoeba model data that have been generated in this work.

c Phenotypes tested as in [72], abbreviations used are: RSer: serum resistance, RBil: bile resistance, RLys: lysozym/lactoferrin resistance, Mot: motility, GenTim: generation time. Binarization of data in [72].

d Biological model tested: mouse [73], [72], K: mouse killer (9 or 10 mice killed over the 10 inoculated), NK: mouse non-killer (no mouse lethality or 1–5 mice over the 10 inoculated), *D.discoideum*, G: grazing, GR: grazing resistance.

The FAC thus shows that virulence is strongly associated with grazing resistance.

To identify possible correlations between grazing resistance to protozoa and the presence of virulence genes and other traits, we performed a κ test of correspondences. This analysis indicates that grazing resistance is correlated to the mouse killer phenotype (κ = 0.459; *p* = 0.00998), to the phylogenetic group B2 (κ = 0.497; *p* = 0.00558), to the resistance to serum (κ = 0.479, *p* = 0.00574), to the resistance to lysozyme (κ = 0.349; *p* = 0.0443), to the resistance to the bile (κ = 0.349; *p* = 0.0443) and to the presence of the genes *iroN* (κ = 0.403; *p* = 0.0222), *fyuA* (κ = 0.412; *p* = 0.0203), *irp2* (κ = 0.412; *p* = 0.0203). Both *fyuA* and *irp2* genes belong to the high pathogenicity island (HPI). This 35- to 45 kb stretch of DNA carries a siderophore-mediated iron uptake system named the yersiniobactin locus [47]. To sum up, the κ analyses reveal that pathogenicity of different *E. coli* strains on mouse, the B2 phylogenetic group and the presence of virulence genes are significantly correlated with grazing resistance to protozoa.

### Experimental demonstration of the role of the HPI in the grazing resistance

The IAI51 strain showed a high-virulence phenotype on mouse [48] and *D. dictyostelium*. This strain was thus selected to test experimentally the HPI impact on *D. dictyostelium* that was already suggested from the correlation analysis. Two mutant strains IAI51 were tested for the amoeba grazing: a IAI51 *irp1*- (carrying a deletion covering the *irp1* gene) and the IAI51 pCP1 (IAI51*irp1*- recomplemented with the *irp1* gene on a plasmid). These mutant strains were already tested on a mouse model of the extra-intestinal virulence and have indicated a role of HPI in the mouse lethality [48].

Five replicates of the three bacterial strains (IAI51, IAI51*irp1-*, IAI51pCP1) were plated at a high bacterial population size (10^8^ cell) and three amoeba population sizes (10, 10^2^, 10^3^ cells) (N = 45 plates). The occurrence of the bacterial lysis plaques was assessed six days after plating (Table 2). This experiment was repeated twice. The presence of the *irp1* gene was highly significantly associated with the grazing resistance to amoeba as the deletion of the *irp1* gene made the strain sensitive to the amoeba (grazing) whereas complementation of this mutant by the cPC1 plasmid restored the grazing resistance (three strains model: IAI51/IAI51 *irp1-*/IAI51 pCP1, ddl = 2, χ^2^ = 30.11, *p*<0.0001; two strains model: IAI51 *irp1-*, IAI51 pCP1, ddl = 1, χ^2^ = 20.49, *p*<0.001; two strains model: IAI51, IAI51 *irp1-*, ddl = 1, χ^2^ = 42.71, *p*<0.001).

**Table 2:** Demonstration of the role of the HPI in the grazing resistance.

Strain	IAI51 pCP1	+	+	++
	IAI51 irp-	+++	++++	++++
	IAI51	−	+	−
	606	+++++	+++++	+++++
		1	2	3
		log(number amoeba)		

Bacterial lysis plaques (strains REL 606, IAI51, IAI51 irp-, IAI51 pCP1) observed six days after plating. A density of 10^8^ bacteria was plated with 10, 10^2^ and 10^3^ amoebae. (+) represents the number of replicates with lysis plaques, (−) means no replicate showing plaques.

## Discussion

This study investigated the coincidental evolution hypothesis of virulence factors. In particular, we tested the hypothesis that some bacterial traits favours the survival of *E. coli* when faced to grazing protozoa. A close relationship was observed between the mouse killer phenotype that represents intrinsic extra-intestinal virulence of the bacterial strain [32] and the grazing resistance trait. *D. discoideum* was able to survive and phagocytize *E. coli* strains that (i) are not harboring virulence genes involved in iron capture (*iroN*, *fyuA*, *irp*), (ii) are not resistant to serum, bile, lysozyme/lactoferine, or that do not belong to the phylogenetic group B2. *D. discoideum* cells are specialized phagocytes that essentially feed on a range of bacteria, their natural prey. After adhesion, engulfment of the bacteria in a phagosome is the first step before successive maturation steps involving sequential delivery and retrieval of components to phagosomes where lysosomal hydrolases are delivered to digest bacteria. The association between grazing resistance and the various resistance phenotypes: resistance to human serum, lysozyme/lactoferine, bile, reflects the importance of the capacity of ExPEC cells to protect their membrane against lysosomal hydrolases from amoeba phagosomes. Other bacterial strains are known to resist digestion by amoeba such as *Mycobacterium avium* bacilli [49], *E. coli O157* vero-cytotoxigenic strain [26] (for a review, see [50]). However, mechanisms that determine resistance to grazing amoeba are complex and not fully understood. For instance, we found no correlation between the presence of the capsule K1 and the resistance of bacteria to amoeba grazing. Previous studies showed that this capsule enables *E. coli* to survive in human endothelial cells by preventing lysosomal fusion [51] and in *Acanthamoeba* [52]. Nevertheless even if this capsule enhances bacterial survival it does not seem to interfere with amoeba survival.

This study shows that various ExPEC strains can resist to digestion by amoeba. The percentage of grazing resistant *E. coli* strains that carry virulence factors is greater (76%) than the percentage of grazing resistant *E. coli* strains that do not carry virulence factors (16%). This is consistent with the hypothesis that predation by protozoa can favour bacterial traits increasing the survival but that emerge as virulence determinants in human infections. Genes responsible for such effects can thus be thought of as being maintained by coincidental selection. An earlier study [9] had already established that some intracellular pathogens normally persist in the environment in close association with protozoa and especially with amoeba. King et al. (1988) [10] suggested that grazing resistance to protozoa was an evolutionary precursor of bacterial pathogenicity. This was supported by the finding of predation-mediated variation at the *rfb* virulence locus of *Salmonella enterica* which illustrate the potential impact of protozoan grazing on the origin of bacterial pathogenicity [53]. Recently, Steinberg and Levin [54] showed that the Shiga toxin genes carried by some intestinal pathogenic *E. coli* strains increase bacterial survival in presence of *Tetrahymena pyriformis* indicating that such prey-predator interactions might have driven the evolution of this virulence factor. Similarly, our experiments showed that *E. coli* genes involved in the iron uptake such as *irp* and *fyuA* -that belong to the HPI- and *iroN* were involved in the grazing resistance to *D. discoideum*. Iron is a vital resource for bacteria, necessary for many functions, from respiration to DNA replication. While on earth, it is one of the most abundant elements, its rarity in the biosphere makes it often a limiting resource for microbial growth. Furthermore, in the presence of oxygen, iron is oxidised to the ferric state and form ferric hydroxide that is insoluble in aqueous solution. Many bacteria can exploit *D. dictyostelium* phagocytosis and grow intracellularly [26], [55]. As a counter strategy, many hosts have evolved iron withholding strategies to disable micro-organisms [56]. In *D. dictyostelium*, the transport of iron across the phagolysosomal membrane contributes to pH homeostasis, while at the same time depleting the lysosomal milieu from the iron that is required for bacterial growth. In response to this competition for iron, counter-mechanisms have been developed by microbes to overcome the scarcity of this metal. These mechanisms involve two principal methods: (i) either the synthesis of high iron affinity compounds called siderophores (iron-scavenging agents), or (ii) by a method of direct capture at the bacterial cell membrane [57]. Siderophores are ferric-specific microbial iron chelator compounds which biosynthesis is regulated by the availability for iron in surrounding environment [58]. Bacteria respond to the low-iron environment by increasing expression of iron acquisition system as well as of other various virulence factors [59]. Our IAI51 *irp1-* mutant showed less virulence on *D. dictyostelium* compared to the mutant IAI51 pCP1 and was then consistent with the closed relationship between iron and virulence. For the host, the competition for iron necessitates careful orchestration of the iron regulation to prevent bacteria invasion and optimal iron homeostasis. Thus, some of the virulence factors involved in human disease may have an ecological function within natural communities or even have their origin specifically in successful antipredator adaptation. Other papers [15], [16] showed a possible link between bacterial evolution in the natural environment and their pathogenicity toward animals. They suggested that the pathogenicity genes were selected for alternate ecological functions.

Our grazing resistance experiment showed a strong correlation between lethality in mice and grazing resistance. This is the first demonstration that *D. discoideum* can be used as host model for specifically detecting ExPEC strain virulence. More generally, previous studies [60], [61] reported a high correlation between virulence as estimated from its resistance to *D. discoideum* grazing and as apparent in a mammalian infection model. Our results suggest that for ExPEC *E. coli* strains, similar mechanisms determine virulence in mammalian systems and amoeba. This relation is corroborated by correlation between resistance to lysozyme or serum and grazing resistance in our experiments. From a bacterial point of view, the similarities between *D. discoideum* and mammalian cells include membrane trafficking, endocytosis, phagocytosis and exocytosis pathways [45], [55]. Moreover, *D. discoideum* shares its chemotactic capacity with leucocytes. Thus *D. discoideum* grazing looks like killing by macrophages [62], [63]. The amoeba host model (*D. discoideum*) has already been used with a variety of bacterial species: *L. pneumophila* [55], *Pseudomonas aeruginosa* [61], *Vibrio cholerae* [64], *Aeromonas* spp (*A.salmonicida* and *A.hydrophila*) [65]. The relevance of this model is not only based on the observation that many pathogens show a low species specificity but also on that a large range of hosts will encounter the same bacteria in their natural environment. The *D. discoideum*–*E. coli* model is a very simple and powerful system. Using a simple plating assay, *D. discoideum* forms a phagocytosis plaque on a lawn of non-pathogenic bacteria but does not on a lawn of pathogenic ones. The virulence of the bacteria used may thus be extrapolated from the ability *of D. discoideum* to survive and grow in their presence [61], [66]

The fact that free living amoebae graze on bacteria has important implications from ecological and evolutionary viewpoints [67]. Ecological interactions are often grouped according to the net effect that each species act on its partner (positive, negative or null ones). Such definitions of species interactions are then classified as either competitive, antagonistic or mutualistic. But the interaction between bacteria and amoebae shows many facets. The interactions between *E. coli* and *D. discoideum* are complex and the balance between conflict and interest may be delicate. *E. coli* can find food inside *D. discoideum* but at the expense of a substantial risk to be digested. Our death vs growth inhibition experiment showed that once inside *D. Dictyostelium*, a struggle for life begins. Bacteria may exploit amoeba phagocytosis to find key nutrients, but depending on the strain characteristics, either the bacteria resists amoeba digestion and kills *D. discoideum* (Figure 2 (B) fourth row) or it is digested by *D. discoideum* (Figure 2 (B) third row). As soon as bacteria and amoeba show sufficient common interest they may both sustain themselves (Figure 2 (B) first and second rows), but if selfish interests prevail common interest, the harmonious relationship stops and the struggle for life begins until the death of either one of the partners. If bacteria are able to resist predation, invading amoebae presents a number of potential advantages: the amoebae protect them against adverse environmental conditions and may even act as vector for bacteria toward new resources. So even if their defense against predation is not perfect, bacteria may be selected to let themselves be phagocytized notwithstanding the risk. Such interactions that are partly antagonistic, partly mutualistic have been called ‘dangerous liaisons’: partners team up but nevertheless relentlessly pursue their private interests [68]. Amoeba could be not only protective niches under stressful conditions or vehicles but also trojan horses –e.g amoebal vesicules containing bacilli could be inhaled by humans and bacteria may then infect them [14], [69].

Another ecological factor such as abundance seems to be related to grazing resistance. Our study demonstrated that the relative population size of bacteria/amoeba may play a key role in grazing resistance (Figure 1). When the relative population size of pathogenic bacteria to amoeba is sufficiently high no plaque lysis was observed (10^7^ bacteria for 100 amoeba cells or 10^8^ bacteria for 1000 amoeba cells). If relative population size of bacteria to amoeba is lower than a certain threshold however we observed plaques and no grazing resistance. Many species of bacteria including *E. coli* K-12 MG1655 [70] and O157:H7 [71] were shown to regulate expression of certain genes in response to change in population size. In the primary (the gut) and secondary (in natura) *E. coli* habitats, bacteria and amoeba populations coexist. In natura, they are dispersed in patches in soil. Clarholm [42] observed that amoebae were as abundant as 10^5^ individuals per gram of soil. Their numbers may increase up to 20-fold after four days thus becoming 2.10^6^ individuals per gram of soil. Amoeba may have been documented to cause a decline of prey bacteria in nature from 10^8^ to 10^5^ cells per gram of soil. Thus, in the soil, the relative amoeba/bacteria population size is likely to be high enough to permit the amoeba to have a great impact on bacterial population. Nevertheless, the establishment of a community depends on the history of the site, the order in which the species arrived, what kind of interactions develop among species and patch quality. All these factors determine if such a community would achieve a permanent state of equilibrium [42]. The primary *E. coli* habitat, the gut form a complex ecosystem with various commensal amoeba species coexisting with more than 500 bacterial species totalizing 10^10^ to 10^11^ cells per gram of content of the large intestinal. Commensal bacterial strains may coexist with amoeba in the gut and our experiments showed that whatever the relative amoeba/commensal bacteria population sizes amoeba may survive and grow.

### Concluding remarks

In this paper, we argued that ecological factors are associated with the evolution of bacterial traits responsible of virulence. Our observations support the coincidental hypothesis for the evolution of virulence: the capacity to resist grazing by protozoa manifests itself in a human host as increasing virulence. Amoeba and bacteria are in a closed relationship in their different habitats (in natura vs in the gut).

The use of *D. discoideum* to detect ExPEC strain virulence has several benefits. The simplicity of the experimental protocol and its reproducibility surpasses those of mammalian systems. Furthermore, numerous genetic tools allow the use of different well known *D. discoideum* mutants to study the mechanisms behind pathogenicity (endocytosis, phagocytosis), the sensitivity to pathogens or to identify genes involved in virulence. Despite some limits (temperature culture of 24°C, simple cell), *D. discoideum* may prove a fruitful model system for preliminary studies before conducting experiments with mammalian host models.

## Material and Methods

### Strains and culture conditions

#### Bacterial strains

The collection of *E. coli* strains (Table 1) was previously described [72], [73]. These strains were isolated from extraintestinal infections and from feces of healthy humans. They represent 10 commensal, 21 ExPEC and three laboratory adapted strains.

Commensal strains belong mainly to the phylogenetic group A and B1 whereas ExPEC are mainly from the B2 phylogenetic group. One pathogenic strain belongs to the D phylogenetic group.

An *E. coli* HPI mutant and its complemented isogenic strain were tested for *D. discoideum* grazing: the IAI51 *irp1-* mutant (with the *irp1* inactivated gene) and the IAI51 pCP1 mutant (with the recomplementation of the *irp1* gene on plasmid pCP1). The HPI-encoded yersiniobactin system of the strain was inactivated by insertion of a kanamycin resistance gene cassette into the *irp1* gene coding for the yersiniobactin protein HMWPI. These strains were tested in a mouse model of extra-intestinal virulence [48].

The laboratory adapted strain B/r (Pasteur Institute) is a standard food for *D. discoideum*. We also used *E. coli* K-12 MG1655 and B REL606 (Richard Lenski, Michigan State University) strains which are also phagocytized by *D. discoideum* and harmless.

#### Bacterial culture conditions

For all the experiments and all the strains, an overnight culture in 10 ml of HL5 medium (5 g/l proteose peptone, 5 g/l thiotone E peptone, 10 g/l glucose, 5 g/l yeast extract, 0.35 g/l Na_2_HPO_4_, 7 H_2_O, 0.35 g/l KH_2_PO_4_, pH 6.5) with 130 rpm/min agitation was prepared in a 50 ml Falcon tube, incubated at 37°C. On the day of the experiment, the culture was centrifugated (20 000 rpm/min; 20 min), washed once, and resuspended in MCPB (1.42 g/l Na_2_HPO_4_, 1.36 g/l KH_2_PO_4_, 0.19 g/l MgCl_2_, 0.03 g/l CaCl_2_, pH 6.5). Cells were then plated on 5.5 cm diameter plates of HL5 agar.

#### Amoeba strain and culture conditions

The amoeba *D. discoideum* AX3, an axenic strain was used in all the experiments. Amoeba was grown in 10 ml of liquid HL5. Cells from mid-logarithmic cultures were then centrifugated (2000 rpm/min; 7 min) and washed once with MCPB.

For all experiments, a volume of 300 µl was plated. It corresponds to the minimum volume covering all the Petri dish (5.5 cm diameter).

### Assays

#### Relative population size effects

Bacterial strains used in these experiments were the pathogenic strain 536 and the non pathogenic strain B REL606.

In the first part of this experiment, the bacterial population size effect was tested from 10^4^ to 10^8^ bacterial cells faced to 100 amoebas. In the reverse experiment, the amoeba population size effect was tested from 10^2^ to 10^6^ amoebae cells faced to 10^8^ bacteria. Relative population size for both experiments were 10^2^, 10^3^, 10^4^, 10^5^, 10^6^ bacteria cells/amoebae.

A volume of 300 µl bacterial culture was plated on HL5 agar Petri dishes and allowed to dry for 20 min under a sterile air flow. The same volume of amoeba culture was added on these plates and allowed to dry for 20 min under a sterile air flow. Plates were covered with a parafilm and incubated for six days at 24°C. Three replicates were performed for each experiment. Plates were screened at day three and day six to assess the occurrence of bacterial lysis plaques formed by *D. discoideum*.

#### Death versus growth inhibition

In the case where no bacterial lysis was observed, it cannot be distinguished between killing or growth inhibition of amoebae. Another experiment was thus performed in the same conditions than above with 10^8^ bacterial cells (REL606 or 536 strains) and 10^2^ amoebae. Plates were examined two and six days after plating. Three replicates were performed. To disentangle death and growth inhibition, the layer was transferred from plates to a liquid MCPB medium and amoeba and bacterial cells were screened with Bac-live Kit that colors viable cells in green fluorescent and membrane damages in red [26]. This experiment was also done with the strains B/r and IAI51.

#### Grazing resistance

All the collection strains were used in this experiment. Bacteria and amoebae were plated with the same protocol as for the relative population size experiment. This experiment was performed with two different bacterial population size: low (10^4^ cells) and high (10^8^ cells) population size. Each bacterial population size was associated with three amoeba population size: 10, 10^2^, 10^3^ cells. Three replicates were run for the entire collection strains and each population size combination; five replicates were run for the IAI51 mutants.

Plates were checked for the occurrence of bacterial lysis plaques formed by *D. discoideum*, three and six days after plating for the low and high bacterial population size, respectively.

This experiment was repeated two times with the strains IAI51 and mutants, IAI52, B/r, RS218, 536 and REL606.

### Statistical analysis

For the relative population size experiment, a χ^2^ test was applied to test for the effects of various factors on amoeba's growth: strains effect (536 *vs* REL606), relative population size effects and the interaction between strain and relative population size.

For the grazing experiment, to investigate associations among possible grazing resistance and the different phenotypic and genotypic bacterial traits, a factorial analysis of correspondence (FAC) was performed using R software and a binary table with 31 lines corresponding to the collection of strains and 28 columns corresponding to the origin of the strain (pathogen or commensal), the four *E. coli* phylogenetic groups, 13 virulence factors, serum resistance, lysozyme/lactoferrin resistance, bile resistance, motility, growth rate, mouse killer (mouse lethality in a septicaemia model), mouse non-killer [72], grazing resistance and grazing. The “grazing resistance” phenotype corresponds to the plates where we did not observe any lysis plaque, for the three replicates and for all amoebae densities. The “grazing” phenotype corresponds to the plates where we observed lysis plaques for at least one replicate for each amoeba population size or for all three replicates with one or two of the three amoeba densities. For each column, we coded each strain with a binary code: present = 1, absent = 0. The influence of the various factors on grazing resistance was tested with a κ test of correspondence.

For the IAI51 mutants, the presence of the *irp1* gene was tested with a χ^2^ test.
